# Dietary habits after a physical activity mHealth intervention: a randomized controlled trial

**DOI:** 10.1186/s40795-023-00682-4

**Published:** 2023-02-02

**Authors:** Linnea Sjöblom, Stephanie Erika Bonn, Christina Alexandrou, Anna Dahlgren, Helén Eke, Ylva Trolle Lagerros

**Affiliations:** 1 Division of Clinical Epidemiology, Department of Medicine (Solna), Karolinska Institutet, Stockholm, Sweden; 2 Center for Obesity, Academic Specialist Center, Stockholm Health Services, Stockholm, Sweden

**Keywords:** Adults, Diabetes mellitus, Type 2, Exercise, Diet, Smartphone

## Abstract

**Background:**

A healthy diet and a sufficient amount of physical activity are important factors to reduce complications of type 2 diabetes. Diet and physical activity are associated behaviours. Individuals who are physically active have also been shown to have healthier eating habits than sedentary individuals. We aimed to evaluate the indirect effect of a smartphone-based physical activity intervention on dietary habits in patients with type 2 diabetes.

**Methods:**

We performed analyses of secondary outcomes in a randomized controlled trial. The active intervention was use of a smartphone application to promote physical activity during 12 weeks. Dietary intake was assessed at baseline and after three months using a validated semi-quantitative food frequency questionnaire comprising 94 items. We analysed changes in the intake of fruit and vegetables, snacks, fibre, whole grains, vitamin C, saturated fat, unsaturated fat and total energy. We also assessed overall dietary habits using a dietary index developed by the Swedish National Board of Health and Welfare. Results were compared between the intervention and control group, as well as stratified by sex within the study groups. Paired t-tests and analysis of covariance were performed.

**Results:**

A total of 181 patients were recruited to the DiaCert-study, whereof 146 patients had complete dietary data and were included in the analyses. Women in the intervention group had a higher fruit and vegetable intake (*p* = 0.008) and a higher dietary index (*p* = 0.007), at three-months compared to women in the control group. They had increased their daily intake of fruit and vegetables by on average 87.4 g/day (*p* = 0.04) and improved their dietary index by on average 0.8 points (*p* = 0.01) from baseline to follow-up. No effect was found in men.

**Conclusions:**

Women, but not men, receiving a smartphone-based physical activity intervention improved their total intake of fruit and vegetables. The transfer effect, i.e. an intervention aimed at promoting one health behavior that facilitates changes in other health behaviors, may differ between the sexes.

**Trial registration:**

ClinicalTrials.gov Identifier: NCT03053336; 15/02/2017.

## Background

The number of individuals with type 2 diabetes is increasing worldwide, with a global prevalence of 8.5% [1]. Consuming a healthy diet rich in fruit and vegetables, fibre and whole grains, achieving sufficient levels of physical activity and maintaining a healthy body weight are cornerstones, both in the prevention of, and to decrease the risk of complications of the disease [1–4].

Combined interventions targeting both physical activity and dietary habits have been shown to improve blood lipid profiles and reduce glycated hemoglobin (HbA1c) in patients with type 2 diabetes, with clinically significant levels up to half a year [5, 6]. However, from these intervention studies, it is not possible to disentangle whether the effect is due to changes in physical activity levels or dietary intake. A high level of physical activity has been associated with other healthy lifestyle behaviors, including a high fruit and vegetable intake, low intake of alcohol, sufficient sleep and low levels of stress [7]. Physically active older adults, have also been shown to have a higher intake of fruits and vegetables than less active older adults [8]. In addition, high levels of moderate physical activity have been cross-sectionally associated with a low intake of added sugar and high consumption of nutritional foods such as fruits and vegetables [9].

An increase in physical activity level has shown to lead to better appetite regulation, although there may be some compensation for energy expended [10, 11]. A systematic review by Donnelly et al. [12] found no consistent evidence that changes in physical activity altered total energy intake or nutrient intake among healthy adults. Also, Amireault et al. [13] examined longitudinal associations between fruit and vegetables intake and moderate-to-vigorous physical activity (MVPA) among participants who survived breast cancer and found no associations between changes in fruit and vegetables intake and changes in levels of MVPA. To the best of our knowledge, there are no longitudinal studies examining prospective changes in diet following changes in activity among patients with type 2 diabetes. Although previous research has shown positive effects of physical activity interventions on dietary habits [7, 14] and cross-sectional observational studies [8, 9] have shown an association between physical activity and diet, it has not yet been established whether dietary habits change as an effect of a physical activity intervention among patients with type 2 diabetes [7, 14–16]. In an attempt to address this issue, we investigated whether a smartphone-based intervention targeting physical activity in patients with type 2 diabetes also would have an effect on dietary habits.

## Methods

### Study design

The DiaCert-study is a randomized controlled clinical trial with two parallel arms. The primary outcome was to evaluate the effect of an app-based step count intervention on MVPA. The study design has been described in detail elsewhere [17]. Here, we conducted analyses of secondary outcomes to investigate the effect of the physical activity intervention on dietary variables at the three-month follow-up. Participants were enrolled between February 2017 and June 2018 in five primary health care centers and one specialized diabetes center in and around Stockholm, Sweden. We completed the data collection in June 2019. The research was conducted at Karolinska Institutet and the study was approved by the Swedish Ethical Review Authority. The trial was registered at ClinicalTrials.gov, Identifier: NCT03053336; 15/02/2017.

### Inclusion and exclusion criteria

Inclusion criteria were: having a diagnosis of type 2 diabetes, ≥ 18 years of age, being able to read and understand Swedish, having access to and being able to use a smartphone. Exclusion criteria were: not being able to walk.

A total of 181 individuals met the inclusion criteria and agreed to participate in the DiaCert-study. In total, 146 individuals had complete dietary data from the food frequency questionnaire both at baseline and at three-month follow-up.

### Participants and recruitment

Participants were recruited in collaboration with nurses and physicians at the participating health care centers. Patients interested in participating, and who fulfilled the inclusion criteria, were contacted by study personnel via telephone. They received more information about the study and were invited to an information meeting in person at the different health care centers. During the meeting, the participants provided their written informed consent to enroll into the study. The participants responded to a comprehensive questionnaire regarding lifestyle habits and were randomized 1:1 to intervention or control group. The randomization was performed separately for women and men in blocks of ten within each primary health care center.

### Study assessments

Dietary intake was assessed through a validated semi-quantitative food frequency questionnaire (FFQ) [18]. Overall dietary habits were assessed by using a dietary index questionnaire developed by the Swedish National Board of Health and Welfare routinely used for clinical practice [19].

#### Food frequency questionnaire

The FFQ included 94 questions, of which 85 questions assessed intake of foods and beverages and the remaining questions assessed alcohol intake. Supplement intake was not assessed. Participants filled in how often, on average, they consumed each item in the FFQ, by answering the question “How much on average do you eat/drink the following items”. There was no specified time frame. The consumption of foods and beverages was then calculated using average portion sizes and converted into nutritional data for daily intake in grams, including total energy, macro- and micronutrients, based on data from the Swedish Food Agency [20].

In this study, we analysed intake of vitamin C, fibre, saturated fat, unsaturated fat, whole grain, and total energy intake, as they are markers of a healthy diet [2–4, 21]. We also retrieved the total intake (grams per day) of vegetables, fruits, and snacks from the FFQ. The “vegetable” variable summarized intake of: lettuce, cabbage, cauliflower, broccoli, tomato, paprika, spinach, peas, onion, carrot, mixed vegetables and legumes. The “fruit” variable summarized intake of: orange, apple/pear, banana, berries and other fruits, and the “snacks” variable summarized intake of: coffee bread, biscuits, pastries, chocolate, sweets, ice cream and chips. A combined variable for “fruit and vegetables” was also created. Intake of fruit and vegetables was categorized as an intake < 400 g/day or ≥ 400 g/day. The cut-off was chosen based on the World Health Organization (WHO) recommendation of a daily fruit and vegetable intake of at least 400 g [22].

#### Dietary index

We also assessed overall dietary intake using the dietary index [17]. The index comprised four questions on the general intake of: 1) vegetables, 2) fruit and berries, 3) seafood and 4) coffee bread, sweets, chips, other snacks or sugar sweetened beverages. Each answer gave 0–4 points depending on how often the food was consumed. This was then calculated to a summary score (0–12 points). The index provided an estimation of whether the dietary guidelines from the Swedish Food Agency were met. The dietary index has been validated against several more comprehensive dietary surveys in the Swedish population [23]. Unhealthy dietary habits were graded as low adherence to recommendations (0–4 points), moderate adherence (5–8 points), or high adherence (9–12 points) to the recommendations.

#### Characteristics

Participant characteristics, including age, sex, level of education and smoking habits, were assessed in the questionnaire at baseline. Body weight was measured to the nearest 0.1 kg in light clothes without shoes using an electronic scale, and height was measured to the nearest 0.5 cm without shoes using a measuring stick. Body mass index (BMI) was calculated as kg/m^2^. Fasting glycated hemoglobin (HbA1c) was measured in venous blood samples and analysed at Karolinska University Hospital laboratory.

Physical activity was measured using the Actigraph wGT3x-BT accelerometer. Participants wore the accelerometer on their non-dominant wrist during all hours for seven days. Accelerometer data processing was performed using R-package GGIR, version 2.0–0 [24]. Default settings were applied, including: MVPA cut-point 100 milligravity (m*g*), 5 s epoch-length and a valid week was defined as four days (with at least one weekend day). Participants were divided into three groups based on how they adhered to WHO’s recommendations for physical activity of at least moderate-intensity [25]. Groups were: below recommendation (< 150 min/week), within recommendation (150–300 min/week) or above recommendation (> 300 min/week).

#### Intervention

The intervention group received a smartphone-based physical activity intervention (daily steps) using the DiaCert app [17] during a period of twelve weeks. The features of the app were based on behavior change techniques such as goal setting, feedback and self-monitoring of behavior to increase physical activity [26]. Details of the intervention have been described previously [17]. In brief, together with study personnel, each participant set their own personal daily step goal that ranged between 1,000 and 10,000 steps based on their usual activity. Daily steps were recorded in their smartphone and automatically presented in the app. A positive push message with feedback saying “Well done, you have reached your step goal” was sent in the app every day when the step goal was achieved. Participants could also visually follow their progress in the app in with circles that “filled-up” during the day towards their individual daily goal and a check mark when the goal was reached. Further, a bar chart was also displayed in the app, presenting the daily steps from all days as well as the average number of daily steps over the last seven days. Data on daily steps was shared with the study personnel. No information about diet was included in the app. In addition to physical activity, the app also displayed baseline HbA1c value. The app did not include any other information, for example weight control or other diabetes issues. The control group continued with their usual care.

### Statistical analysis

Descriptive statistics were conducted using mean ± SD for continuous variables and n (%) for categorical variables. Differences in baseline characteristics between study groups were examined using independent t-tests and chi-squared tests, respectively. Wilcoxon signed-rank test was used to examine differences in whether the participants reached the recommended 400 g of daily fruit and vegetable intake, as well as to examine changes in dietary index categories, between baseline and follow-up. In order to investigate the intervention effect on dietary variables, i.e. differences in improvement between intervention and control at three-month follow-up, analysis of covariance (ANCOVA) was used with baseline intake as a covariate. Differences between groups over time stratified by sex and adjusted for baseline dietary intake were also performed using ANCOVA.

Sensitivity analysis adjusting for baseline MVPA in three categories, i.e. below recommendation (< 150 min/week), within recommendation (150–300 min/week) or above recommendation (> 300 min/week) were also performed, as there was a statistically significant difference in MVPA between the groups at baseline. Results remained virtually the same (data not shown). Paired samples t-tests were used to analyse changes in dietary intake within groups, stratified by study group, as well as stratified by study group and sex. Statistical significance was defined as *p*-values < 0.05. We conducted the statistical analyses using STATA, version 15.1 (Stata Corporation, College Station, TX, USA).

## Results

Baseline characteristics of all participants included in this study are presented in Table 1. The average age of the participants was 60.7 years ± 11.1 (ranging from 31.4 to 84.8 years), the majority were men (65%), and 47% had a university degree. The mean BMI was 30.2 kg/m^2^, with 45.2% having a BMI above 30 kg/m^2^. Almost half, 46.6%, of participants reported an intake of fruit and vegetables above 400 g/day. According to the dietary index, 5.0% were classified as having low adherence (0–4 points), and 35.5% were classified as having high adherence (9–12 points) to the recommendations.

**Table 1:** Characteristics of all participants at baseline (*n* = 146), and stratified by intervention and control group

**Characteristics**	**All** (*n* = 146)	**Intervention **(*n* = 72)	**Control** (*n* = 74)	*p*-value^1^
	Mean ± SD	Mean ± SD	Mean ± SD	
BMI, Body Mass Index (kg/m^2^)	30.2 ± 5.5	29.9 ± 5.7	30.4 ± 5.3	0.57
HbA1c, Glycated hemoglobin (mmol/mol)^2^	53.5 ± 15.2	54.0 ± 13.3	53.0 ± 11.9	0.64
Dietary index^3^	7.7 ± 1.9	7.9 ± 1.9	7.5 ± 1.9	0.21
	**n (%)**	**n (%)**	**n (%)**	
**Age**				0.12
< 50 years	26 (17.8)	15 (20.8)	11 (14.7)	
50–59 years	38 (26.0)	21 (29.2)	17 (23.0)	
60–69 years	52 (35.6)	27 (37.5)	25 (33.8)	
> 70 years	30 (20.6)	9 (12.5)	21 (28.4)	
**Sex**				0.96
Men	95 (65.1)	47 (65.3)	48 (64.9)	
Women	51 (34.9)	25 (34.7)	26 (35.1)	
**Education level**^**2**^				0.90
Elementary school, ≤ 9 years	15 (10.4)	8 (11.4)	7 (9.5)	
High school, 10 to 12 years	61 (42.4)	30 (42.9)	31 (41.9)	
University, ≥ 12 years	68 (47.2)	32 (45.7)	36 (48.7)	
**Dietary index**^**3**^				0.19
Low adherence (0–4 p)	7 (5.0)	3 (4.3)	4 (5.6)	
Moderate adherence (5–8 p)	84 (59.6)	37 (52.9)	47 (66.2)	
High adherence (9–12 p)	50 (35.5)	30 (42.9)	23 (28.2)	
**Current smoker**^**3**^	17 (11.8)	9 (12.7)	8 (11.0)	0.75
**MVPA (min/week)**^**4**^				0.04
< 150	49 (36.3)	17 (25.8)	32 (46.4)	
150–300	45 (33.3)	27 (40.9)	18 (26.1)	
> 300	41 (30.4)	22 (33.3)	19 (27.5)	

*MVPA* moderate-to-vigorous physical activity

^1^Differences between groups using two-tailed t-test (continuous variables) and chi2 test (categorical variables). Statistical significance is defined as *p* < 0.05

^2^*n* = 144

^3^*n* = 141

^4^*n* = 135

**Fig. 1:**
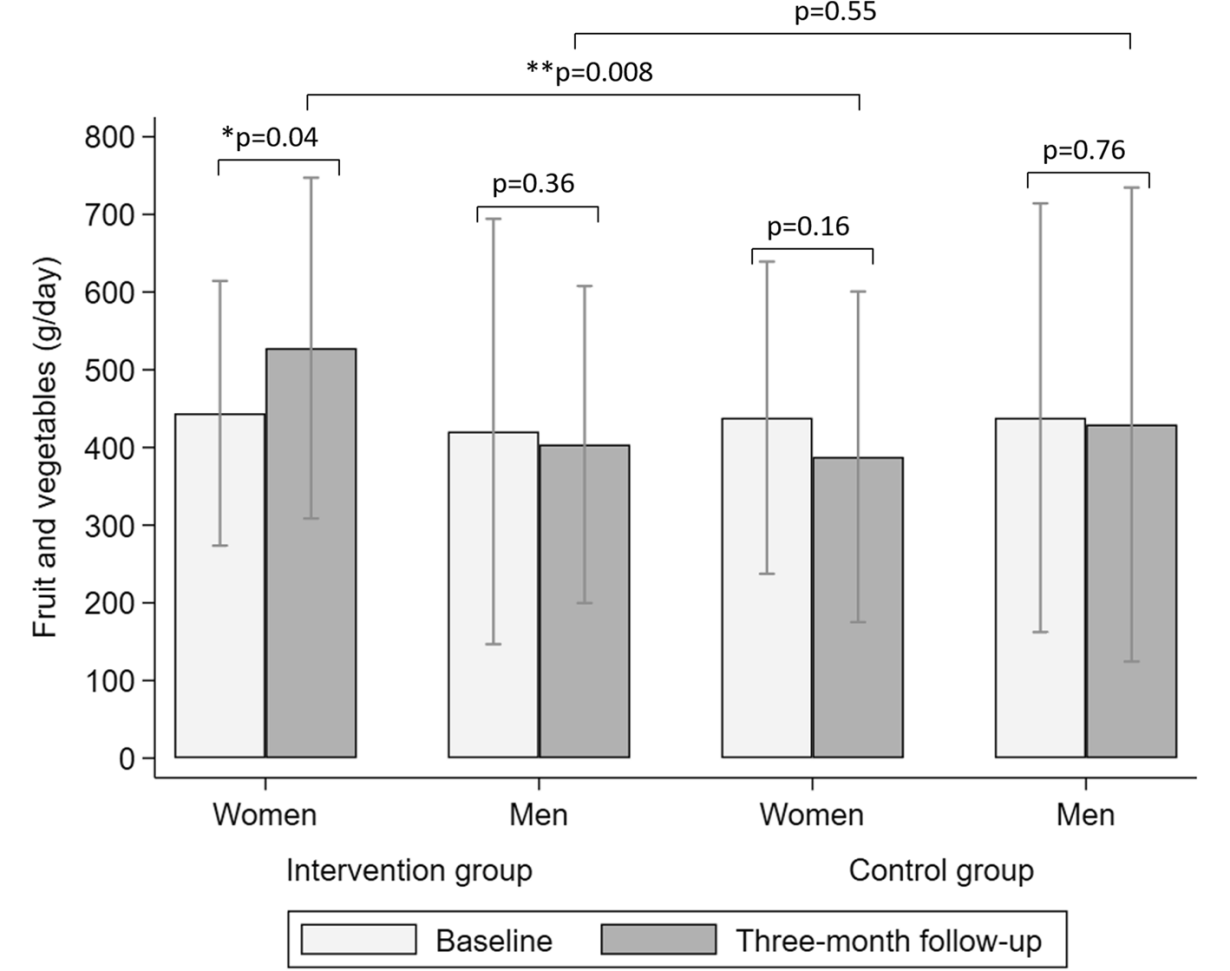
Mean ± standard deviation for total intakes of fruit and vegetables, between baseline and the three-month follow-up, stratified for women and men in the intervention and the control group. *Differences between baseline and the three-month follow-up, stratified by sex and intervention and control group using paired samples t-tests. **Differences between intervention and control group at the three-month follow-up stratified by sex using analysis of covariance with baseline as covariate. Statistical significance is defined as *p* < 0.05

The results from the ANCOVA showed no difference in dietary variables at three-months, when comparing the intervention and the control group, adjusted for baseline dietary intakes, see Table 2. However, in the ANCOVA analysis stratified by sex and adjusted for baseline intake of fruit and vegetables, women in the intervention group had a significantly higher (*p* = 0.008) total intake of fruit and vegetables (528 ± 219 g/day) after the intervention period, compared to women in the control group (388 ± 213 g/day), see Fig. 1.

**Table 2:** Comparison between baseline and the three-month follow-up, stratified by intervention and control group

Characteristics	Intervention			Control			
	All (*n* = 72)			All (*n* = 74)			
	Baseline	3-month	Within group	Baseline	3-month	Within group	Between groups
	Mean ± SD^1^	Mean ± SD	*p*-value^2^	Mean ± SD	Mean ± SD	*p*-value^2^	*p-value*^3^
Dietary index^4^	7.9 ± 2.0	8.1 ± 1.8	0.46	7.5 ± 1.9	7.5 ± 1.9	0.74	0.31
Dietary variables							
Fruits and vegetables (g/day)	436 ± 249	447 ± 216	0.69	439 ± 251	415 ± 275	0.29	0.29
Vegetables (g/day)	256 ± 172	258 ± 168	0.92	236 ± 172	235 ± 170	0.95	0.70
Fruit (g/day)	180 ± 160	189 ± 126	0.62	203 ± 150	182 ± 166	0.29	0.45
Fibre (g/day)	27 ± 11	27 ± 10	0.71	27 ± 10	25 ± 13	0.22	0.50
Vitamin C (mg/day)	124 ± 68	122 ± 54	0.86	121 ± 71	115 ± 68	0.35	0.47
Snacks (g/day)	26 ± 21	26 ± 21	0.96	32 ± 38	32 ± 35	0.97	0.51
Saturated fat (g/day)	30 ± 13	29 ± 13	0.41	29 ± 12	29 ± 13	0.73	0.53
Unsaturated fat (g/day)	49 ± 20	48 ± 18	0.40	46 ± 21	45 ± 19	0.48	0.65
Whole grains (g/day)	63 ± 42	60 ± 40	0.45	61 ± 35	57 ± 34	0.12	0.70
Energy (kcal)	2081 ± 727	2004 ± 665	0.23	1965 ± 644	1971 ± 805	0.80	0.43
	n (%)	n (%)		n (%)	n (%)		
Fruits and vegetables			0.64			0.49	
< 400 g/day	40 (55.6)	38 (52.8)		38 (51.4)	41 (55.4)		
≥ 400 g/day	32 (44.4)	34 (47.2)		36 (48.7)	33 (44.6)		
Dietary index			0.65			0.22	
Low adherence (0–4 p)	3 (4.5)	1 (1.5)		4 (5.6)	4 (5.6)		
Moderate adherence (5–8 p)	35 (52.2)	37 (55.2)		48 (66.7)	42 (58.3)		
High adherence (9–12 p)	29 (43.3)	29 (43.3)		20 (27.8)	26 (36.1)		

^1^*SD* Standard deviation. Statistical significance is defined as *p* < 0.05

^2^Differences between baseline and the three-month follow-up, stratified by intervention and control group using paired samples t-tests (continuous variables) or Wilcoxon signed-rank test (categorical variables)

^3^Differences between intervention and control group at the three-month follow-up using analysis of covariance

^4^*n* = 139

When analysing fruit and vegetables separately using ANCOVA among women, there was no difference (*p* = 0.15) in total intake of vegetables in the intervention (284 ± 132 g/day) and control group (211 ± 119 g/day), but the total increase of fruit was significantly higher (*p* = 0.04) in the intervention group (244 ± 149 g/day) compared to the increase in the control group (184 ± 165 g/day). The total intake of vitamin C, followed the same trend, with a greater increase (*p* = 0.02) in the intervention group (139 ± 57 mg/day) compared to the control group (98 ± 48 mg/day). Similarly, they also had a significantly higher (*p* = 0.007) mean dietary index score (8.9 ± 1.6) at the three-month follow-up, compared to women in the control group (7.6 ± 1.4). No differences in the ANCOVA analysis, adjusted for baseline dietary intakes, between the intervention and control group were seen among men.

There was no statistical difference in the dietary variables within the groups at the three-month follow-up, compared to baseline (see Table 2). The average intake of fruit and vegetables in the intervention group was 436 g/day at baseline and 447 g/day at follow-up. The control group reported an average intake of 439 g/day of fruit and vegetables at baseline and 415 g/day at follow-up. When stratified by sex, the total daily intake of fruit and vegetables increased with 87.4 g/day (*p* = 0.04) among women in the intervention group (see Fig. 1). Women also had a significantly higher mean dietary index score (0.8 points; *p* = 0.01) at follow-up, compared to baseline. No changes between baseline and follow-up assessments were seen among men.

## Discussion

The results of our study showed that women receiving a smartphone-based physical activity intervention had a statistically significant increased dietary index score, an increased daily intake of total fruit and vegetables and total vitamin C intake after the intervention, compared to women in the control group. This increase was not seen among men. We did not find an effect of the intervention on any other variables of dietary intake (fibre, wholegrains, snacks, saturated fat, unsaturated fat, or total energy), neither did we find an effect on the primary outcome of the intervention, i.e. on MVPA (unpublished data).

Fleig et al. have suggested that physical activity and dietary habits in cross-behavioral associations appear to be positively related to each other [27], i.e. higher levels of physical activity are associated with healthier dietary intake. The theory behind this is that interventions aimed at promoting one health behavior change may also facilitate other health behavior changes, the so-called transfer effect [14, 28]. In line with our results, prior research has shown that interventions targeting physical activity have a positive effect also on dietary habits, including fruit and vegetable intake [7, 14]. However, not all studies have found changes in dietary intake after physical activity interventions [15, 16]. To the best of our knowledge, there are no studies examining dietary changes after a physical activity intervention in patients with type 2 diabetes.

Among older adults with pre-diabetes, Halliday et al. examined whether spontaneous dietary changes occurred after a twelve-week program of resistance training [29]. While participants had a lower intake of total energy, sugar, carbohydrates, sweets and desserts, they also had a lower intake of fruit and vegetables after the exercise program. The authors suggested that the reduced intake of fruit potentially could have been an effect of media portraying fructose as harmful without distinguishing between fructose present in fruit and that in added sugar. The latter is in contrast to our results of an increased fruit and vegetable intake among women in the intervention arm, while we saw no effect on intake of snacks.

Why we found an effect of the physical activity intervention on fruit and vegetable intake within women, but not men, warrants discussion. In Sweden, the average intake of fruit and vegetables has been reported to be 360 g/day in women and 310 g/day in men [30]. Our population comprising patients with type 2 diabetes, reported a relatively high intake of fruit and vegetables at baseline with 46.6% reporting an intake > 400 g/day, which is in accordance with the WHO dietary recommendations [22]. The high fruit and vegetable intake in our study population may reflect that we have a group that has regular follow-ups with their diabetes nurse and, in contrast to the general population, receive dietary advice on a regular basis.

Women have been shown to be more likely to consume a sufficient amount of fruit and vegetables, while men were more likely to perform adequate physical activity [31]. Also, in a diabetes population similar to ours, women consumed more fruit and vegetables, legumes, vegetables oils and added sugar, whereas men consumed more starchy food, soft drinks and alcoholic beverages [32]. An explanation for the change in dietary variables seen among women in our study may be that it is usually they, especially in their age category (60 + years), who have the main responsibility for both planning and preparation of the food in the home. Thus, women taking part in an intervention may find it easier to change eating habits than men may, simply because they more often than men are in charge of the grocery shopping.

Overall, our study population was adherent to the Swedish Food Agency’s dietary guidelines, with only 5.0% classified as having low and 35.5% classified as having high adherence. This indicates better adherence compared to the general Swedish population, where 20% of adults have been classified as having low adherence, and only 10% high [19]. A reason for this could be that most patients with type 2 diabetes are educated to know which foods that affects blood sugar levels, and therefore to a high degree have healthy diets. However, it could also be due to social desirability bias, i.e. an overestimation of desirable variables and an underestimation of undesirable variables to defend one’s social image [33]. This in turn would lead to an overestimation of healthy dietary habits and thereby an inflation of the dietary index score in our population. Women may be more affected by social desirability than men [34], which may explain the difference in the results between women and men in our study. A high adherence at baseline may have affected the outcome at the three-month follow-up, i.e., if the patients already had healthy dietary habits, they may not have wanted, or needed, to change their diet. They may even have been told by their dietician to be restrictive with their fruit intake, to prevent high blood sugar.

The randomized controlled study design is a strength of our study. It is also an advantage that we have included both women and men and therefore were able to stratify by sex. However, the relatively small number of participants is a limitation. The recruitment of participants from several different primary health care centers located in areas with varying socioeconomic status, increased generalizability. However, the inclusion criteria of being able to communicate in Swedish is a limitation as that might have led to a selection of patients excluding those who recently immigrated, and thereby a lack of ethnic diversity in our sample. This may limit the generalizability of our results. On the other hand, a high proportion (36%) of the study participants were recruited from a health center located in an area with low socioeconomic status and high immigration.

Potentially, those who were already interested in their general health where more prone to participate in the study too. Nevertheless, the mean HbA1c, BMI, and age of our participants were comparable to that of patients with type 2 diabetes in Swedish primary care [35]. The proportion of men included in our study (65%) was slightly higher than the proportion of men with type 2 diabetes in Sweden (57.9%) [36]. In a systematic review, Pagoto et al. highlighted that men, especially older men, may be more difficult to recruit in lifestyle weight loss interventions than women [37]. Thus, it is an advantage that we have recruited a high proportion of men. New technology, such as using an app to change lifestyle behaviors, may have been particularly appealing for men. Another reason for the high proportion of men recruited to our study may be due to the fact that almost 60% of the Swedish patients with type 2 diabetes in primary care are men [38].

It is a limitation that we, due to the study design, could not blind participants to the group allocation. This is always a challenge in lifestyle interventions. Nonetheless, the active intervention only focused on physical activity and there was no information about diet in the app or during the intervention. Blinding of study personnel is a way to prevent bias in ascertainment of outcomes. In this study, only laboratory personnel analysing HbA1c was blinded. On the other hand, anthropometric measurements like objectively measured body weight should be less inclined to be biased, and calculation and analyses of dietary variables from the FFQ were performed in the same way regardless of study group, which reduces the risk of bias.

The interpretation of absolute nutritional values from the FFQ should be done with caution. Although the FFQ included 94 different food items, it is also limited to the food items listed in the questionnaire and to the use of standard portions sizes. There may have been an effect of the intervention on other dietary variables than those we selected and examined in our study. Another limitation is that the recall period for the FFQ was not specified when participants were asked how often, on average, they consumed each food and drink item in the FFQ. This may limit the ability of the FFQ to capture short term dietary intakes, and change over three months. However, it is a cognitive challenge to assess the frequency of food consumption over a wide time frame [39], and respondents, even if asked to specify dietary intake over a longer time period, may still remember and report the most recent intake. The memory about actual consumptions of foods erodes after only a few days [40]. Moreover, we have only studied short term dietary change. Future studies are needed to examine the long-term effects of a physical activity intervention on dietary habits.

The variables chosen from the FFQ were selected as markers of a healthy diet, for example a diet rich in fibre and whole grains, which has been shown to improve glycemic control [2–4]. Further, a reduced intake of saturated fat in favour of unsaturated fat is beneficial to improve blood lipids [21]. According to national guidelines, it is recommended for patients with type 2 diabetes to keep blood lipids low to avoid complications and prevent cardiovascular disease [41]. Nonetheless, the answers to the FFQ are based on the respondents’ memory of how they usually eat with a possibility of both under- and overreporting of certain food items [42]. Almost half, 45%, of the participants had a BMI > 30 kg/m^2^. Previous studies have shown that there is a higher risk of underreporting food intake among individuals with overweight or obesity [43]. On the other hand, intake of food items that generally are considered healthier (such as fruit and vegetables) often tend to be overestimated [43]. This should not have affected our results, since participants were randomized to intervention or control group, thus creating equal distributions of BMI in both groups. However, it may have influenced our sex-stratified results. In a previous systematic review [44] which examined sex differences in nutrient intake in validations of FFQ against a reference method, it was found that the nutrient intake was significantly more often overestimated in women compared to men.

## Conclusions

Our findings indicate that using a smartphone-app to promote daily physical activity may improve intake of total fruits and vegetables among women, at least in the short run. As this was not seen among men, our results suggest that there might be sex differences in terms of the transfer effect, i.e. the phenomenon where an intervention targeting one health behavior facilitates changes in other health behaviors.

## Data Availability

The datasets analysed during the current study are not publicly available due to ethical restrictions, but are available from the corresponding author on reasonable request.
